# An Updated Case Report of a Long‐Term Survivor With Stage IVB Ovarian Squamous Cell Carcinoma Arising From Mature Cystic Teratoma

**DOI:** 10.1111/jog.70412

**Published:** 2026-07-23

**Authors:** Rina Nagayama, Masao Okadome, Kumi Shimamoto, Yutaka Koga, Kenichi Taguchi, Kazuya Ariyoshi

**Affiliations:** ^1^ Gynecology Service NHO Kyushu Cancer Center Fukuoka Japan; ^2^ Department of Diagnostic Pathology NHO Kyushu Cancer Center Fukuoka Japan

**Keywords:** chemotherapy, cytoreductive surgery, descending colon, squamous cell carcinoma, teratoma

## Abstract

A 68‐year‐old woman visited a gynecological clinic complaining of abdominal distension and pain. A large immobile tumor was palpable in the upper left abdominal area. Computed tomography showed an ovarian tumor measuring 20 cm in diameter anterior to the left kidney. A barium enema revealed that the descending colon was narrow, with a wall that was irregularly transformed by the tumor. Cytoreductive debulking surgery was performed. A histological examination demonstrated squamous cell carcinoma arising from a mature cystic teratoma of the ovary. The descending colon was invaded by cancer cells up to the mucosal tissue, and four lymph nodes were metastatic. After surgery, she received eight cycles of adjuvant chemotherapy. After the last treatment, she has been free of the disease for longer than 18 years. We describe the clinical characteristics of this case and speculate on the reason for the long‐term survival.

## 1 Introduction

Mature cystic teratoma (MCT) is the most common neoplasm of the ovary, occurring in 10%–20% of women. The reported frequency of malignancies in ovarian MCT is 0.17%–2.0%, of which 80% are squamous cell carcinomas (SCCs) [1]. Ovarian SCC arising from MCT typically occurs in postmenopausal women [2] and has a poor prognosis, particularly in the advanced stage [3]. Previously, we reported cases of malignant transformation of ovarian MCT at our institution, including stage IVB ovarian SCC. In this previous report, we briefly described the same case as that in the current report but did not describe macroscopic, microscopic, or imaging findings or evaluate long‐term survival. This particular case has been free of disease for more than 18 years since the previous report was published. Currently, the patient is alive and well and thus appears to be cured. Therefore, we believe that performing an update of this unique case and describing the clinical characteristics in detail would provide useful knowledge. Consequently, we report here this patient with stage IVB ovarian SCC arising from MCT who has survived for a long time.

## 2 Case Report

**FIGURE 1:**
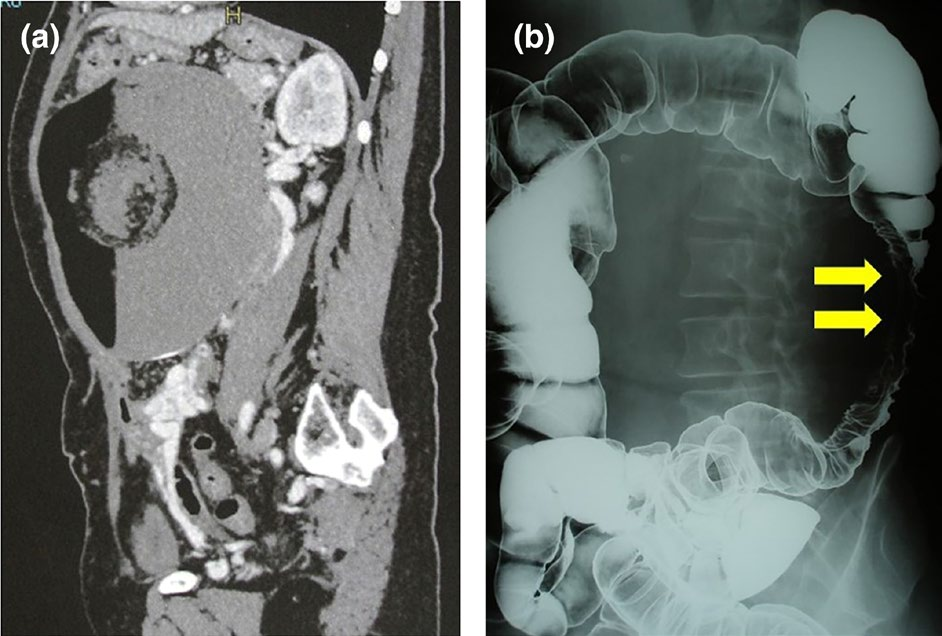
Computed tomography and barium enema of the abdomen. (a) A spherical tumor containing liquid was observed anterior to the left kidney. (b) The descending colon was narrow, and its wall was irregularly transformed by the tumor.

A 68‐year‐old woman (gravida 3 para 3) visited a gynecological clinic with complaints of abdominal distension and pain. Gynecological cancer was suspected in computed tomography (CT), and thus she was referred to our hospital. A large immobile tumor was palpable in the upper left abdominal area. CT showed an ovarian tumor measuring 20 cm in diameter anterior to the left kidney and attached to the descending colon. The left ovarian vein was distended. Although the site was unusual, the tumor was suspected to originate from the left ovary. A barium enema revealed that the descending colon was narrow, with a wall that was irregularly transformed by the tumor (Figure 1). Tumor markers were elevated, including SCC antigen (132.0 ng/mL), carcinoembryonic antigen (23.9 ng/mL), cancer antigen (CA) 19–9 (492 U/mL), and CA125 (113 U/mL).

**FIGURE 2:**
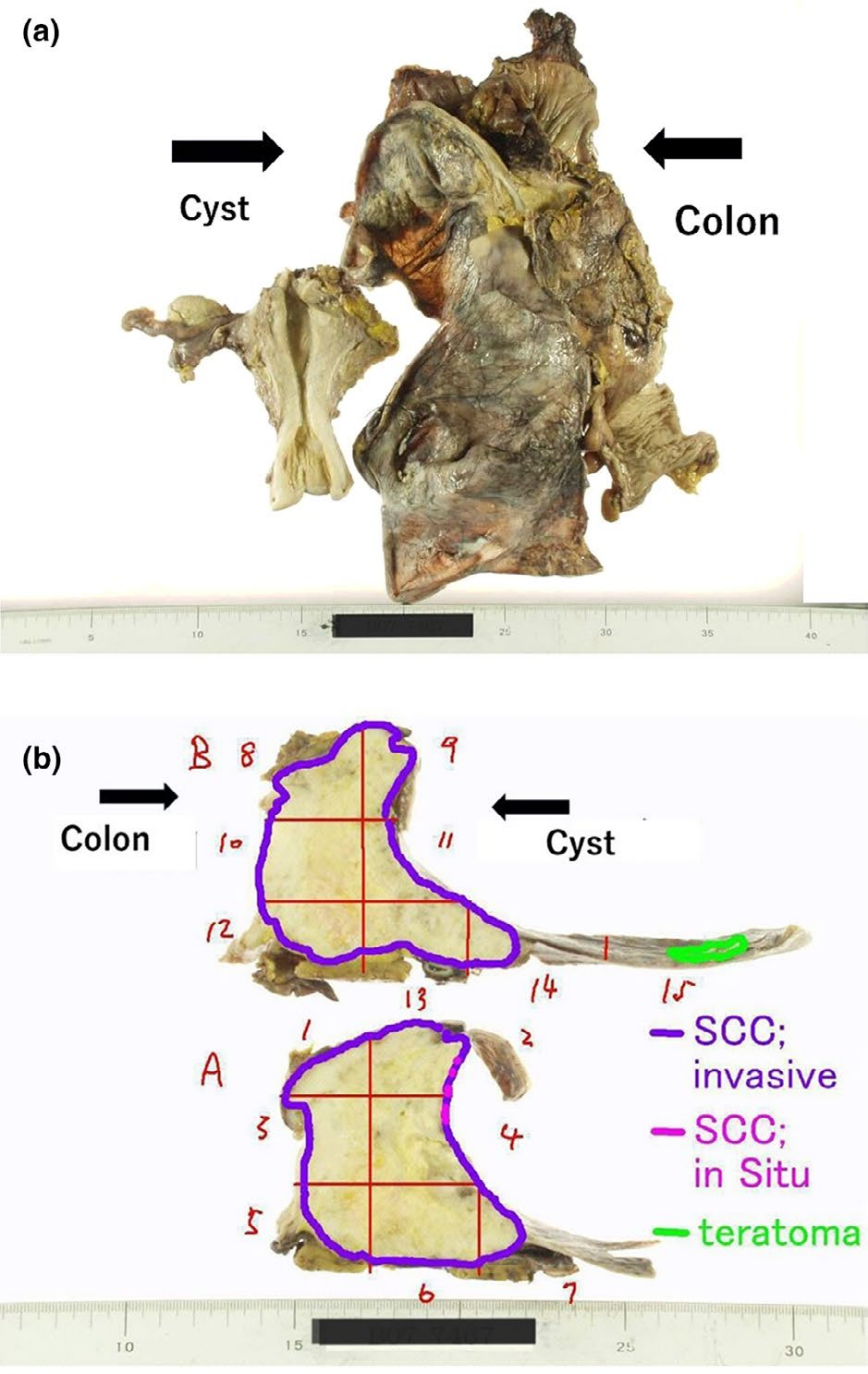
Macroscopic characteristics of the tumor. (a, b) Hair and fat tissues are present in the irregular solid part of the tumor, which had invaded the mucosa of the descending colon.

She underwent a laparotomy, which showed a large tumor of the left ovary invading the descending colon. Macroscopic 100% cytoreductive debulking surgery was performed, including total abdominal hysterectomy, bilateral salpingo‐oophorectomy, omentectomy, appendectomy, descending colon resection and anastomosis, paraaortic artery lymph node dissection, and biopsies of the descending colon mesenteric lymph node and inferior mesenteric artery lymph node. Ascites and peritoneal dissemination were absent. Several lymph nodes were slightly swollen, round, approximately 5 mm in diameter, and white. The main tumor of the left ovary was not ruptured. A part of the tumor had invaded into the descending colon wall and was encapsulated by the outside and inside parts of the descending colon wall. The tumor contained hair and fat tissue (Figure 2). Enlarged or suspicious lymph nodes were removed selectively on the basis of an intraoperative inspection.

**FIGURE 3:**
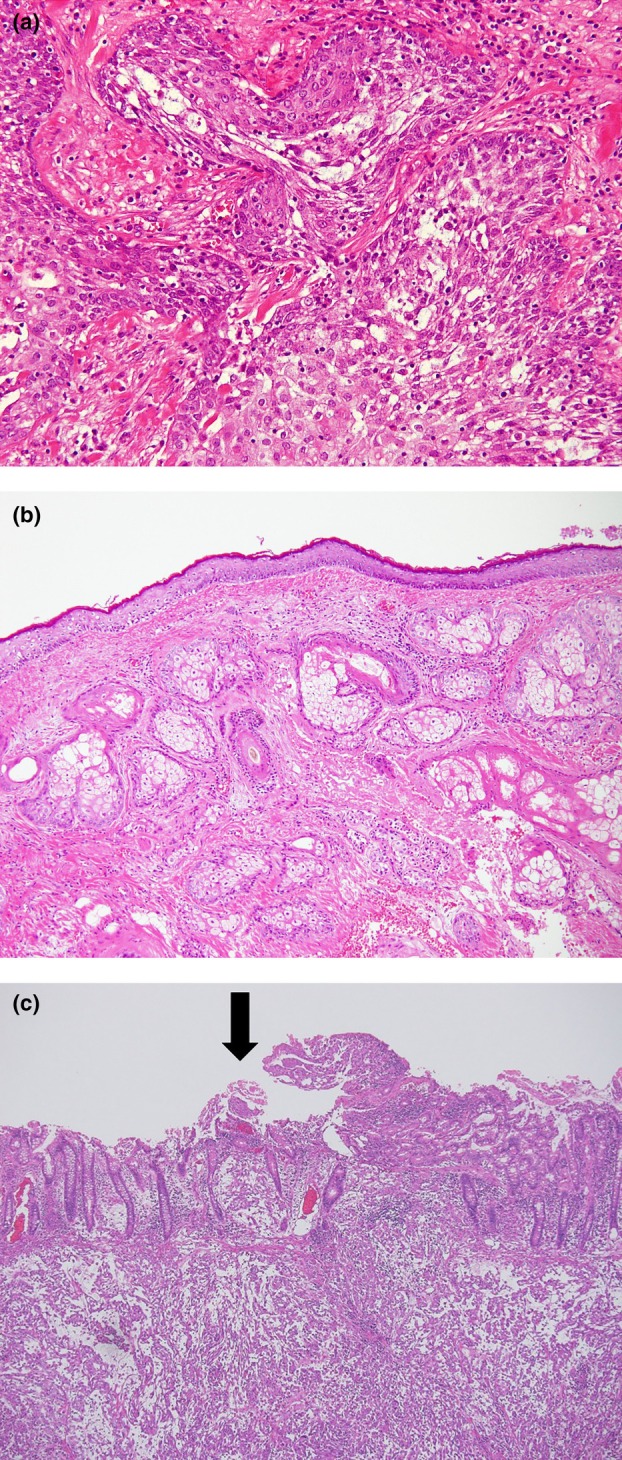
Microscopic characteristics of the tumor. (a) The tumor is composed of atypical squamous cells (squamous cell carcinoma, ×400). (b) A mature cystic teratoma can be seen focally (teratoma, ×100). (c) Invasion of the descending colon mucosa by cancer cells (mucosal invasion, ×100).

A histological examination showed SCC arising from ovarian MCT. The tumor penetrated the mucosal tissue of the descending colon. Of the 12 lymph nodes, 4 were metastatic and included the right para‐aortic, left para‐aortic, mesenteric, and upper renal hilum lymph nodes (Figure 3). Washing cytology of the peritoneal cavity was negative. These findings indicated an International Federation of Gynecology and Obstetrics (FIGO) stage (2014) of IVB and TNM classification (UICC 8th edition) of pT1c2N1M1b. The evidence suggesting stage IVB was mucosal invasion to the descending colon. She received postoperative adjuvant chemotherapy (paclitaxel, 175 mg/m^2^, carboplatin, area under the curve = 5, tri‐weekly, total of 8 courses). After the last treatment, she showed no recurrence of disease for more than 18 years.

## 3 Discussion

Malignant transformation of MCT is rare, and its prognosis is generally poor at advanced stages. Abdominal pain, the presence of an abdominal mass, and abdominal distention are the most common symptoms of this disease. Congcong et al. reported 5‐year overall survival rates of 85.8%, 39.1%, 26.2%, and 0% for stages I, II, III, and IV, respectively [3]. They reported that tumor size is an important factor contributing to the differential diagnosis between a malignant tumor and MCT. They showed that SCC arising from an MCT was larger than an MCT. The mean size of 37 SCCs derived from MCT was 152.3 mm, and the cut‐off size between benign and malignant tumors was 99 mm [3].

Tumor markers are useful for predicting SCC arising from an MCT. Hackethal et al. examined the serum concentrations of different antigens and found that 86.5% (45/52) of patients had high SCC antigen, 71% (36/51) had high CA125, 77% (30/39) had high CA19‐9, and 67% (16/24) had high carcinoembryonic antigen concentrations. They observed no correlations between tumor marker concentrations and the FIGO stage but did observe an association between adverse outcomes and higher concentrations of SCC antigen and CA125 [2].

In this case, the location of invasion was beyond the pelvic cavity and the tumor had invaded the mucosal tissue. The diagnosis of stage IVB was based on the transmural invasion to the descending colon. If there was no transmural invasion, the mode would have been close to stage II because of the difference in the location of invasion.

In this case, macroscopic 100% cytoreductive surgery was performed. Gadducci et al. analyzed data from 10 reports and found that the tumor stage and debulking status were the strongest prognostic variables [4]. Congcong et al. reviewed published data (from 435 cases) regarding SCC transformation in ovarian MCT from 1977 to 2016 at Qilu Hospital, Shandong University [3]. Regarding surgical treatment, hysterectomy improved survival rates, whereas lymphadenectomy did not. Omentectomy was associated with a better prognosis after adjusting for the FIGO stage, whereas no differences in mortality were observed between fertility‐sparing and radical surgery in patients with stage IA or IC.

Our case received postoperative adjuvant chemotherapy (paclitaxel and carboplatin regimens), which can improve the survival of patients with advanced stages. Chemotherapy with platinum is associated with a better prognosis than other regimens. In contrast, radiotherapy and chemoradiotherapy do not improve survival [3]. Bleomycin, etoposide, and cisplatin therapy is the standard chemotherapy in germ cell tumors. Ovarian SCC arising from MCT is derived from germ cell tumors, but the site of malignant transformation is categorized as epithelial cancer. Therefore, chemotherapy with platinum is considered an appropriate regimen.

The prognosis for malignant transformation of MCT is poor, particularly for advanced stages, with 5‐year overall survival rates of 26.2% for stage III and 0% for stage IV. Regarding the early stage, in a case of stage IC2, the main tumor ruptured before laparotomy, and the prognosis was poor even after paclitaxel and carboplatin therapy [5]. Tomita et al. reported a retrospective study of seven SCC cases of malignant transformation of an MCT between January 2000 and December 2017. Of these patients, some presented with atypical ascites cytology (*n* = 2) or intraperitoneal dissemination (*n* = 2), some underwent suboptimal surgery (*n* = 2), and some died within 13 months after surgery (*n* = 5) [6]. Hackethal et al. described that the prognostic factors for ovarian carcinoma include capsular invasion, rupture, tumor dissemination, ascites, adhesions, and additional invasive tumor types [2].

In the present case, peritoneal cytology was negative, dissemination did not spread to the abdominal cavity, and the area of the tumor that invaded the descending colon was encapsulated by the outside and inside of the colon wall. The tumor was resected without rupture, and enlarged or suspicious lymph nodes were resected completely followed by eight cycles of paclitaxel and carboplatin therapy. The surgical findings in our case are unique and thus may have been the underlying reason for the good outcome. Fortunate spread patterns, complete surgery, and adequate adjuvant chemotherapy may be associated with successful treatment, even for advanced disease stages. We consider that the most effective factor for the good outcome was the unique encapsulated spread pattern. This pattern enabled us to resect the primary focus and macrometastatic foci. Potential residual lesions could be micrometastases in lymph nodes that were not removed. Eguchi et al. reported that chemotherapy was more effective for lymph nodes than for the gross primary lesion [7]. Adjuvant paclitaxel and carboplatin therapy may be effective when the remaining tumor cells are present only in the lymph nodes. Novel drug treatments, such as immune checkpoint inhibitors, could improve the prognosis of ovarian SCC arising from MCT that has spread to the abdominal cavity.

## Author Contributions

**Yutaka Koga:** visualization, writing – review and editing. **Kumi Shimamoto:** writing – review and editing. **Rina Nagayama:** conceptualization, methodology. **Kazuya Ariyoshi:** supervision, validation. **Masao Okadome:** conceptualization. **Kenichi Taguchi:** visualization, writing – review and editing.

## Disclosure

The authors have nothing to report.

## Ethics Statement

The authors have nothing to report.

## Consent

The authors have nothing to report.

## Conflicts of Interest

The authors declare no conflicts of interest.

## Data Availability

The data that support the findings of this study are openly available in NHO English Article Database at https://nho.hosp.go.jp/research/publication.html.

## References

[1] S. Y. Wu , A. Giannini , M. Girardo , A. Schmitt , J. F. Magrina , and K. Butler , “Malignant Transformation of Squamous Cell Carcinoma in Mature Cystic Teratoma of the Ovary: A Systematic Review and Meta‐Analysis of Data,” Gynecologic and Obstetric Investigation 19 (2024): 1–13. 10.1159/000542672 PMC12129424 39561732

[2] A. Hackethal , D. Brueggmann , M. Bohlmann , et al., “Squamous‐Cell Carcinoma in Mature Cystic Teratoma of the Ovary: Systematic Review and Analysis of Published Data,” Lancet Oncology 9 (2008): 1173–1180. 19038764 10.1016/S1470-2045(08)70306-1

[3] L. Congcong , Z. Quing , Z. Siying , et al., “Squamous Cell Carcinoma Transformation in Mature Cystic Teratoma of the Ovary: A Systematic Review,” BMC Cancer 19 (2019): 217–228. 30866852 10.1186/s12885-019-5393-y PMC6417039

[4] A. Gadducci , M. E. Guerrieri , and S. Cosio , “Squamous Cell Carcinoma Arising From Mature Cystic Teratoma of the Ovary: A Challenging Question for Gynecologic Oncologists,” Critical Reviews in Oncology/Hematology 133 (2019): 92–98. 30661663 10.1016/j.critrevonc.2018.10.005

[5] T. Shimada , A. Higashijima , A. Fukushima , et al., “Malignant Transformation From Mature Cystic Teratoma of the Ovary,” Journal of Obstetrics and Gynaecology Research 45 (2019): 1957–1960. 31215124 10.1111/jog.14043

[6] Y. Tomita , T. Saito , M. Okadome , et al., “A Glint of Hope for Treatment of Advanced Malignant Transformations of Ovarian Mature Cystic Teratomas: A Retrospective Analysis of 9 Cases,” Journal of Gynecologic Surgery (2020): 1–5, 10.1089/gyn.2019.0134.

[7] T. Eguchi , Y. Kodera , H. Nakanishi , et al., “The Effect of Chemotherapy Against Micrometastases and Isolated Tumor Cells in Lymph Nodes: An In Vivo Study,” In Vivo 22 (2008): 707–712. 19180995

